# Editorial: Insights in integrative physiology: 2021

**DOI:** 10.3389/fphys.2023.1186581

**Published:** 2023-03-27

**Authors:** James T. Pearson, Jacqueline K. Phillips, Geoffrey A. Head

**Affiliations:** ^1^ National Cerebral and Cardiovascular Center, Suita, Japan; ^2^ Department of Physiology, Monash Biomedicine Discovery Institute, Monash University, Clayton, VIC, Australia; ^3^ Macquarie Medical School, Macquarie University, Sydney, NSW, Australia; ^4^ Baker Heart and Diabetes Institute, Melbourne, VIC, Australia

**Keywords:** metabolic, endocrine, immune, cardiovascular, respiratory, renal, reflex, neural

The past decade has seen significant advancements in the field of Integrative Physiology. This series, Insights in Integrative Physiology 2021, published in Frontiers in Physiology, aims to highlight these developments, providing a comprehensive overview of what has been achieved and future challenges, in order to inform, inspire and provide direction and guidance to researchers in the field. The series is unified by the exploration of interconnected metabolic, endocrine, immune, cardiovascular, respiratory, renal, and neural systems that work together to maintain homeostasis. Each paper offers a distinct perspective on this topic. And while the papers are organised thematically in this editorial, it should be apparent to readers that it is challenging to examine each system independently of the others.

As a perfect start to the Research Topic, the commentary provided by Curtis provides a perspective on the importance of integrative physiology education, seen through the lens of angiotensin II research over the last 30 years. The author argues that the field of physiology has shifted towards the cellular, molecular and genetic, resulting in inadequate understanding of whole body integrative physiological function. The author contends that while students have extensive knowledge of the molecular biology of angiotensin signalling, they do not know about the outcome of that signalling, which is crucial for understanding the interrelated and complementary neural, cardiovascular, renal, and endocrine elements. The take home message is that integrative physiology should be given more attention to ensure its significance and appreciation are reinforced in both education and research.

The paper by Simões-Alves et al. reviews the cardiometabolic effects resulting from postnatal hyperlipidic diets in protein-restricted subjects, and the epigenetic repercussions occasioned by a phenomenon termed nutritional transition. The authors describe a trend over the last decade whereby maternal protein undernutrition during pregnancy and lactation followed by a high-fat diet has been linked to the development of cardiometabolic diseases in the offspring. Nutritional transition is associated with growth restriction, increased blood pressure, dyslipidaemia, and insulin resistance, as well as sympathetic overactivity, hypertension, and disruptions in respiratory control. They examine the epigenetic mechanisms proposed to be involved in these cardiometabolic effects including changes in DNA methylation and increased transcription of genes involved in the regulation of blood pressure and insulin sensitivity. The paper highlights how our understanding of complex epigenetic mechanisms may help understand cardiovascular disease risk with intergenerational and transgenerational implications.

Original research in this Research Topic also examines the consequences of protein restriction during sensitive windows of postnatal growth and how this can also predispose to cardiovascular disease. Oliveira Ferreira et al. demonstrate that the peri-pubertal period in male rats is a sensitive period that is vulnerable to the programming of autonomic dysfunction and cardiovascular remodelling in adulthood. Moreover, this research team revealed that a brief exposure to the low protein diet and subsequent catch-up growth evoked elevated peripheral sympathetic activity (hypertension with increased arterial pressure low frequency power spectral density) and altered redox state in both the brainstem and the heart that led to the onset of cardiac remodelling.

The topic also attracted a number of in-depth reviews covering other aspects of metabolism. Shi and Qiu reviewed some of the newer reports on myocardial energy substrate metabolism, cardiac metabolic remodelling during exercise and cardiac development. Tan and Naylor reviewed how new knowledge of metabolic, protein and transcriptional pathways that occur in breast and prostate cancer can be influenced by modifiable lifestyle factors to change cancer cell physiology to reduce risk and benefit prognosis.

Blood pressure regulation and sodium-water homeostasis are modulated by the mineralocorticoid receptor (MR) through endogenous aldosterone and corticosteroids. For this reason, MR antagonists have received considerable attention as valuable first line therapies for hypertension and heart failure. However, beyond the vasculature, chronic activation of MR in cardiomyocytes is shown by Bienvenu et al. to adversely alter extracellular matrix composition and myocardial inflammatory state, redox balance, intracellular ion composition and apoptosis, leading to increased vulnerability of the heart to ischaemia. This mini-review presents compelling evidence for sex-specific differences in MR signaling involving estrogen receptor interactions that need to be further explored if we are to reduce the greater heart failure burden that exists in women.

Another key process that regulates sodium-water homeostasis is osmoregulation and vital to the regulation of osmolarity is the neuroendocrine supraoptic nucleus (SON) of the hypothalamus. Mecawi et al. provide an overview of the current understanding of the mechanisms that regulate plasma osmolarity within the SON, including osmosensing, transcriptomic plasticity of during long term hyperosmolality and the somatodendritic release of neuropeptides in response to osmotic challenge.

Touching on several themes within the Research Topic Badoer reviews the important contributions of circulating cytokines and locally-induced inflammation in the brain and overactivation of the sympathoadrenal and central renin angiotensin (RAS) systems in the promotion and potentiation of autonomic dysfunction in heart failure. The work highlights likely reasons why anti-inflammatory therapies have often been ineffective in preventing central inflammation in heart failure and the challenges associated with identifying targets within the brain and effective methods of delivery of therapeutic compounds across the blood-brain barrier.

A relatively new area of research is the influence of the gut microbiome on the immune and the cardiovascular systems. Abboud et al. examined the effect of altering post-natal gut microbiota in the spontaneously hypertensive rat (SHR) on the development of the immune system and the development of hypertension. The method used was cross fostering which changed two major bacteria taxa to those of the normotensive strain, also a pro inflammatory immune cell population was reduced in the spleen and aorta as was the blood pressure of cross-fostered SHR. However, the changes in the microbiome of the normotensive control rats that were cross fostered by SHR, did not change blood pressure in these animals. The mechanism likely involves the changes to the gut biome since it is known that faecal transplants from normotensive rats also lowers blood pressure in SHR (Toral et al., 2019).

Linking the immune and neural systems, Bognara et al. examine the relationship between a centrally mediated autonomic reflex and inflammation. The Bezold-Jarisch reflex is a well characterised response to stimulation of afferents in the cardiopulmonary region that evokes bradycardia, hypotension, and apnoea. Typically, a selective serotonergic 5-HT3 receptor agonist, phenylbiguanide can be used experimentally to activate the reflex. In order to determine whether evoking this reflex could modulate the immune system, the authors measured cytokines in spleen, heart, blood and hypothalamus during lipopolysaccharide induced inflammation. Interestingly, the reflex reduced plasma levels of tumour necrosis factor and interleukin 6 levels in spleen compared to controls but no changes in other cytokines in other tissues were observed. While the precise mechanism is not known, the study confirms the novel interaction between the nervous system and inflammation, helping us to understand this important relationship.

Exploring vascular pathology, Li et al. review the potential of curcumin, a compound found in turmeric, as a therapeutic agent for hypertension and vascular remodelling. They highlight its ability to inhibit protein tyrosine kinase activity, suppress protein kinase C activity, and block protein kinase and mitogen-activated protein kinase (MAPK) pathways. Curcumin has also been shown to block platelet-derived growth factor (PDGF) and the epidermal growth factor receptor (EGFR), both of which are linked to signaling cascades associated with the proliferation and migration of vascular smooth muscle cells. However, the authors also acknowledge challenges in the use of curcumin as a therapeutic agent, such as poor bioavailability, inconsistencies between animal and human studies, potential side effects, and a lack of evidence for drug interactions. Their review highlights the need to bridge the gap between molecular mechanisms and physiological outcomes in order to unlock the full translational potential of research.

The work by Vieira et al. examines the impact of aerobic physical training on hypertensive hearts, and specifically how training impacts cardiac autonomic control, morphology, and functionality. Using the SHR as a model, the authors show that untrained animals had greater sympathetic contribution to basal heart rate and increased morphological parameters when compared to untrained normotensive rats (WKY), as well as lower coronary bed reactivity and reduced left ventricular contractility in response to increased flow or beta-adrenergic agonist administration. Twelve weeks of swim training resulted in an improved in all but the cardiac morphological parameters. The authors conclude that aerobic physical training could reduce arterial pressure in patients with hypertension through integrative systemic adaptations.

In the brief research report of Vernail et al., the authors utilise gold standard arterial pressure radiotelemetry in mice to investigate for the first time how the non-psychoactive cannabinoid compound cannabigerol (CBG) affects blood pressure following acute administration. These first experiments reaffirm that CBG is indeed a potent inhibitor of sympathetic activity to the peripheral vasculature and heart through its high affinity for inhibitory presynaptic α2-adrenergic receptors, inhibiting noradrenaline release and thus lowering blood pressure and heart rate. The cardiovascular actions of CBG warrant further investigation considering the increasing interest in the use of CBG as a treatment for various neurological conditions and cancer.

Vasopressin, also known as antidiuretic hormone, is typically associated with its role in regulating urine concentration. However, it also has a key role in maintaining homeostasis across many of the systems included in this Research Topic. Proczka et al. present a novel perspective on vasopressin, suggesting that it may play a role in modulating ventilation through both direct actions as a circulating hormone and by acting as a central neurotransmitter *via* vasopressin V1a receptors within respiratory centres of the brainstem. As many factors including hypovolemia, hypotension, glycaemic state, exercise, and angiotensin II all are stimulants of vasopressin release, the evidence for respiratory modulation is carefully assessed and future perspectives to test this hypothesis of a role in fine-tuning respiratory homeostasis are provided.

The kidneys are the final mediator of responses to sodium challenge, but this is regulated by integrated cardiovascular, sympathoinhibitory and natriuretic responses. Having previously demonstrated that Gαi2 subunit proteins in the CNS are necessary to preserve sodium balance, and that these proteins are upregulated in the hypothalamic paraventricular nucleus (PVN) when there is a high intake of salt, Moreira et al., aimed to determine the role of Gαi_2_ proteins in response to central *versus* peripheral sodium challenge and the role of known sodium sensing mechanisms on central Gαi_2_ protein-mediated responses and expression. Using oligodeoxynucleotide (ODN) administration to downregulate brain Gαi_2_, they show that these proteins are essential in mediating the integrated responses to peripheral, but not central, alterations in sodium homeostasis. Their studies further suggest a novel interaction between salt sensitive renal afferent nerves and the upregulation and activation of PVN Gαi2 protein gated pathways which are required to maintain a salt resistant phenotype.

Diabetic nephropathy is a leading cause of chronic kidney disease, with activation of the renin angiotensin aldosterone system playing a leading role as a driver of renal inflammation and fibrosis. However, ACE2, an enzyme that converts angiotensin II into angiotensin 1–7, has renoprotective properties that counteract these effects. In their comprehensive review, Nomura et al. propose that activating this pathway may mitigate kidney inflammation and fibrosis and improve kidney function in diabetic nephropathy. Although several compounds that stimulate ACE2 have been identified, none have progressed to clinical use, presenting a promising opportunity for novel drug development to treat this increasingly prevalent clinical condition.

Several papers in the topic look in depth at centrally mediated physiological reflexes that regulate the cardiovascular system. The PVN for example possesses dual neuroendocrine and autonomic functions and Savić et al. present an overview of its role as a major integrative centre for the regulation of blood pressure and blood pressure variability.

In another review article, Miki et al. consider context-specific changes in response to behaviour and external stress in sympathetic nerve activity (SNA) and the shifts in arterial baroreflex loops that accompany these differences. The arterial baroreflex loop is a CNS control system that regulates the blood supply in parallel to individual organs. The review highlights three examples under which the system is challenged (freezing, REM sleep, and treadmill exercise). They show that regionally specific and co-ordinated changes in SNA, heart rate and arterial pressure under these different conditions and resultant behaviours is the result of changes in baroreflex curves, permitting optimised circulatory regulation. From a translational perspective, the authors conclude while these shifts may also occur in the disease state, they are dysfunctional, perturbing their interaction with other regulatory systems and thereby compounding the disease presentation.

The muscle metaboreflex is a centrally mediated physiological response to prolonged exercise, in which the accumulation of excess metabolites in active skeletal muscle activates metabosensitive skeletal muscle afferents. Due to subsequent enhanced sympathetic outflow to the heart and peripheral vasculature, this enables the dynamic matching of changes in left ventricular elastance with peripheral arterial elastance during physical activity. In heart failure, there is a break down in this ventricular-vascular coupling leading to enhanced systemic vasoconstriction and under perfusion of skeletal muscle. Mannozzi et al. hypothesise that under normal conditions, the arterial baroreflex functions to restrain the muscle metaboreflex induced increase in effective arterial elastance in order to maintain muscle perfusion, but that in heart failure, this restraint is attenuated by impaired arterial baroreflex function. The authors elegantly demonstrate this is indeed the case in chronically instrumented canines.

Staying within the theme of supplying energy to active skeletal muscles through the circulatory system, Yamanaka and Waki present an original study focussed on the amygdala, which is an important integrative site for the processing of behavioural, emotional and cardiovascular responses to aversive and non-aversive stressors. The central nucleus is one of the important subnuclei that is a major area involved with the cardiovascular component of the reaction to stress but its role in dynamically changing stimuli has not been investigated. In a major study in rats, the authors examined this topic by recording the blood pressure and heart rate of head restrained rats given dynamically changing appetitive and aversive classical conditioning tasks (Pavlovian procedures). Through a complex series of unpredictive changing events from neutral, aversive to appetitive stimuli, the authors found that the central nucleus is important for adaptively and rapidly regulation of blood pressure in response to emotional conditioned stimuli.

We return now to the gut microbiome, but this time how microbial metabolites and by-products impact the nervous system to modulate thermal sensation, neural conductance, and thermogenesis. In the review by Zhang and Wang, the authors detail recent discoveries that have led to our understanding of how the gut microbiota contributes to the host’s ability to thermoregulate in cold climates. Symbiotic gut microbiota is sensitive to the temperature of the air and can promote non-shivering thermogenesis by the production of bacterial metabolites and such as short-chain fatty acids and secondary bile acids. It is a fascinating example of coevolution of host-microorganism symbionts which help small animals survive in harsh and variable environment.

The final paper we present is the opinion article of Rasia-Filho. They provide a comprehensive overview of evolving techniques used to explore the intricate and diverse characteristics of postsynaptic dendritic spines in regional and cell-specific contexts. High-resolution imaging and single-cell genomics approaches have provided new insights into brain microcircuits and neural plasticity. The author argues that a multilevel analysis combining single-cell transcriptome data, epigenome databases, and detailed neuroanatomy is needed to understand the function of dendritic spines in information processing and connectivity within regional networks. This could include using large-scale nanoscopy and biochemical analysis of protein distributions across dendrites.

In the fast-growing field of Integrative Physiology, major advancements have been made in the last few years, leading to exceptional achievements as we move into the third decade of the 21st Century. This special edition Research Topic, consisting of 26 outstanding submissions, has provided a thorough overview of the current state of the art of the field, highlighting not only the progress made but also the future challenges. The significance of integrative physiology in comprehending intricate homeostatic mechanisms, both in healthy and pathological conditions cannot be overstated and the Research Topic identifies the importance of exploring the underlying mechanisms of proposed therapeutic interventions, be they related to lifestyle, diet, pharmacological or interventional approaches. Furthermore, we must integrate not only at the systems level but also delve into the cellular, molecular, and genetic. This approach is crucial in attaining a thorough understanding of complex physiological processes and implementing effective therapeutics for improved health outcomes.
